# The effect of strengthening nurse practitioners’ competency in occupational health services for agricultural workers exposed to pesticides in primary care units, Thailand: a before-and-after study

**DOI:** 10.3352/jeehp.2025.22.14

**Published:** 2025-04-21

**Authors:** Napamon Pumsopa, Ann Jirapongsuwan, Surintorn Kalampakorn, Sukhontha Siri

**Affiliations:** 1 Department of Public Health Nursing, Faculty of Public Health, Mahidol University, Bangkok, Thailand; 2 Department of Epidemiology, Faculty of Public Health, Mahidol University, Bangkok, Thailand; The Catholic University of Korea, Korea

**Keywords:** Nurse practitioners, Clinical competence, Occupational health services, Primary health care, Thailand, Quasi-experimental study

## Abstract

**Purpose:**

This study aimed to evaluate the effect of the Strengthening Nurse Practitioners’ Competency in Occupational Health Service (SNPCOHS) program. It was hypothesized that nurse practitioners (NPs) participating in the program would demonstrate increased competency in providing occupational health services to agricultural workers exposed to pesticides in primary care units (PCUs) compared to their baseline competency and to a comparison group.

**Methods:**

A quasi-experimental study was conducted between August and December 2023. The 4-week intervention included 5 hours of an e-learning program, 3 hours of online discussion, and 2 hours dedicated to completing an assignment. The program was evaluated at 3 time points: pre-intervention, post-intervention (week 4), and follow-up (week 8). Sixty NPs volunteered to participate, with 30 in the experimental group and 30 in the comparison group. Data on demographics, professional attributes, knowledge, skills, and perceived self-efficacy were collected using self-administered questionnaires via Google Forms. Data analysis involved descriptive statistics, independent t-tests, and repeated measures analysis of variance.

**Results:**

The experimental group demonstrated significantly higher mean scores in professional attributes, knowledge, skills, and perceived self-efficacy in providing occupational health services to agricultural workers exposed to pesticides compared to the comparison group at both week 4 and week 8 post-intervention.

**Conclusion:**

The SNPCOHS program is well-suited for self-directed learning for nurses in PCUs, supporting effective occupational health service delivery. It should be disseminated and supported as an e-learning resource for NPs in PCUs (Thai Clinical Trials Registry: TCTR20250115004).

## Graphical abstract

[Fig f4-jeehp-22-14]

## Introduction

Thailand’s agricultural workforce comprises nearly half of the total workforce, and almost all agricultural production processes rely heavily on pesticides [1]. This corresponds to a high volume of pesticide imports, which nearly doubled between 2000 and 2024 [2]. Pesticide poisoning remains a critical occupational health issue with acute and chronic harmful effects on agricultural workers, with an incidence rate of 9.58 per 100,000 people reported in 2024 [3].

Nurse practitioners (NPs) are the primary providers of these services for agricultural workers exposed to pesticides, and their competency which encompasses professional attributes, knowledge, and skills, directly affects the quality of patient care. However, most NPs have not received specialized training in occupational health nursing [4]. Current evidence indicates that occupational health services in primary care units (PCUs) do not follow occupational health and environmental medicine service standards [5]. Many NPs face significant challenges due to insufficient knowledge and skills in pesticide toxicology, initial diagnostic procedures for referrals, educating high-risk groups based on blood test results, teaching, and screening [6]. Existing occupational health training for healthcare workers is not specifically designed for NPs in PCUs, resulting in a lack of confidence in delivering occupational health services. Strengthening NPs’ competencies—professional attributes, knowledge, and skills—is essential for delivering evidence-based occupational health care and clarifying their roles in PCUs [7].

Self-efficacy theory, a key framework for competency development, suggests that individuals with higher perceived self-efficacy are more likely to demonstrate desired behaviors in practice [8]. Programs based on self-efficacy theory have effectively enhanced nurses’ professional attributes, knowledge, and skills. E-learning has emerged as an effective and flexible method for training NPs without interrupting patient care or requiring time away from work, addressing common barriers such as limited availability and scheduling conflicts [9]. Therefore, strengthening NPs' competencies in interpersonal relationships, care management, integrated healthcare services, and professional accountability can improve their performance in occupational health services [10]. Therefore, this study applied self-efficacy theory to develop and evaluate the effectiveness of the Strengthening Nurse Practitioners’ Competency in Occupational Health Service (SNPCOHS) program, which includes self-study through e-learning, video clips, online discussions, and case study assignments, thus enabling NPs in PCUs to provide effective occupational health services and improve agricultural workers’ health outcomes and well-being.

### Objectives

**Fig. 1.**
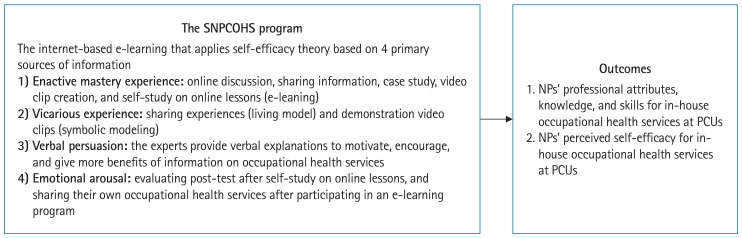
Conceptual framework. SNPCOHS, Strengthening Nurse Practitioners’ Competency in Occupational Health Service; NP, nurse practitioner; PCU, primary care unit.

This study aimed to investigate the effectiveness of the SNPCOHS program in strengthening NPs’ competencies for providing occupational health services to agricultural workers exposed to pesticides in Thailand’s PCUs. The specific objectives were: (1) to assess changes in NPs’ professional attributes, knowledge, and skills in delivering occupational health services; and (2) to examine changes in NPs’ perceived self-efficacy in providing these services (Fig. 1).

## Methods

### Ethics statement

This study was approved by the Ethical Review Committee for Human Research, Faculty of Public Health, Mahidol University (approval no., MUPH 2022-084). Written informed consent was obtained from all participants before data collection, and the study adhered to the principles of the Declaration of Helsinki.

### Study design

This study employed a before and after design, specifically a non-equivalent comparison group pre- and post-test design, with time-based, non-randomized group assignment. A randomized controlled trial was not feasible due to several factors: (1) practical constraints in randomizing NPs in real-world PCU settings; (2) risk of contamination, as NPs may share knowledge and practices; (3) operational limitations in resource-constrained PCUs; (4) stakeholder and policy preferences for timely implementation; and (5) ethical concerns regarding withholding services from high-risk agricultural workers. The study was conducted in accordance with the TREND (Transparent Reporting of Evaluations with Nonrandomized Designs) statement and was registered with the Thai Clinical Trials Registry (TCTR20250115004).

### Setting

Sixty NPs from PCUs in Chiang Rai Province were recruited, with 30 assigned to the comparison group and 30 to the experimental group. Enrollment occurred between August and December 2023. The comparison group completed questionnaires at 3 intervals, each separated by 4 weeks, using Google Forms (Google LLC). The experimental group completed the same questionnaires before the intervention, immediately post-intervention, and at follow-up.

### Participants

**Fig. 2.**
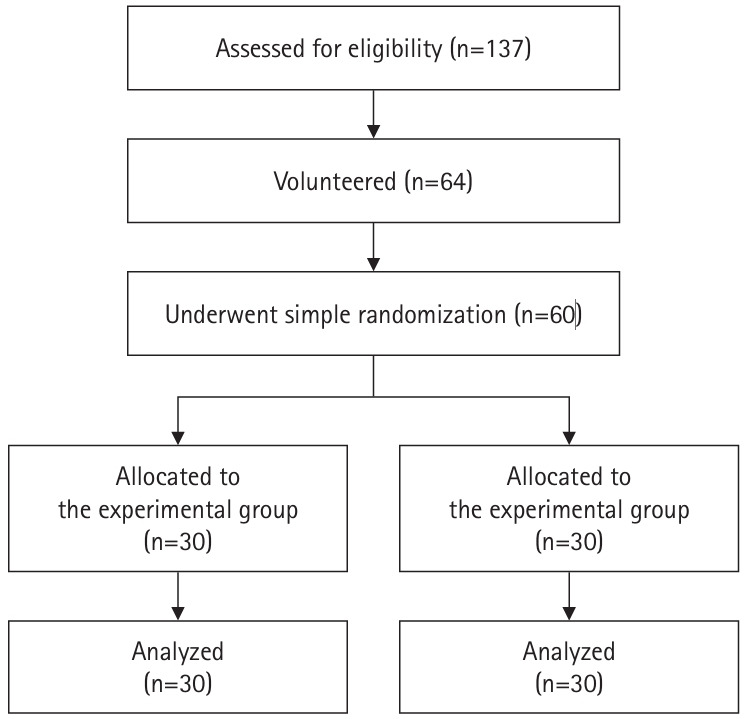
Flow diagram of participants for allocation.

A total of 60 NPs participated, all having at least 1 year of experience working in PCUs in Chiang Rai, Thailand. Participants were computer literate and had internet access. Initially, 64 NPs expressed interest and met inclusion criteria. Simple random sampling was used to select 60 NPs, evenly divided between experimental and comparison groups (Fig. 2). No participants withdrew during the study.

### Interventions

The SNPCOHS program, developed based on a literature review and grounded in the self-efficacy theory by Bandura [8], comprised 10 modules covering key topics such as pesticide fundamentals, occupational health laws, screening and referral, primary care, risk communication, and the role of nurses in occupational health services. A pilot test involved 5 NPs in Phayao Province.

**Fig. 3.**
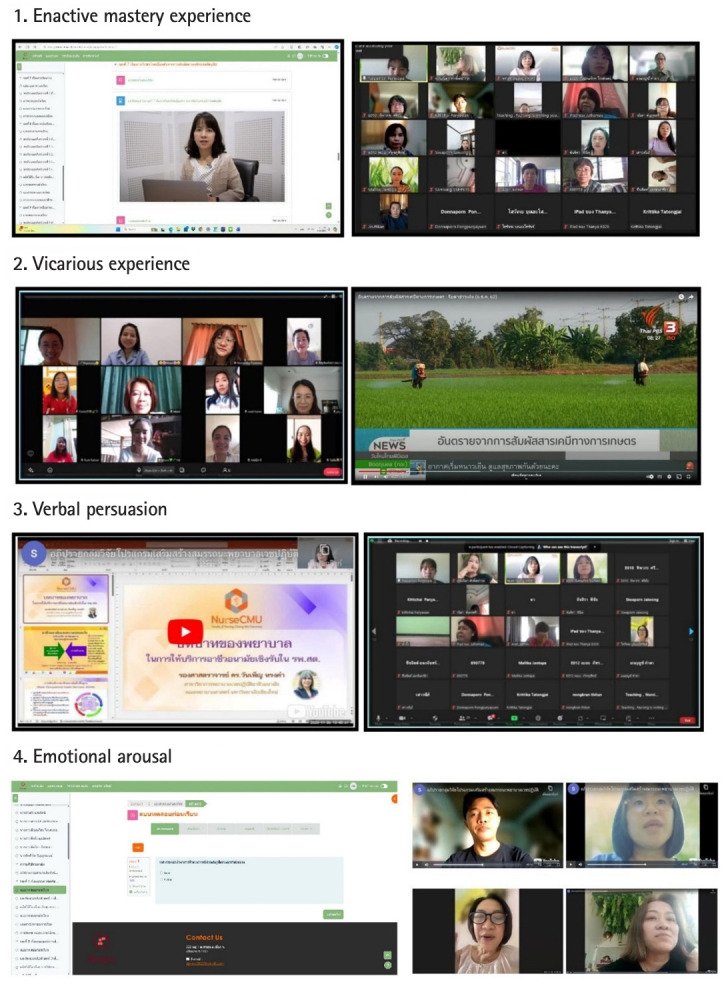
Example of activities applying 4 methods based on self-efficacy theory.

The 4-week program (totaling 10 hours) integrated 4 self-efficacy strategies: enactive mastery, vicarious experience, verbal persuasion, and emotional arousal (Fig. 3). Activities included self-paced e-learning with video clips (Weeks 1–4), post-lesson quizzes (emotional arousal), case-based assignments (Weeks 2–3), and online discussions with peer-sharing and expert feedback (Week 4) (Supplement 1).

### Outcome

The outcomes measured included 4 questionnaire subscales: professional attributes, knowledge, skills, and perceived self-efficacy in providing occupational health services at PCUs for agricultural workers exposed to pesticides (Dataset 1).

### Data sources/measurement

The instrument consisted of 3 parts: Part 1 included demographic variables: age, gender, marital status, highest educational level, length of work experience after completing an NP short course, and work in PCUs. Part 2 assessed professional attributes (10 items, 5-point Likert scale: 5=highest level of competency to 1=no competency), knowledge (18 true/false items), and skills (21 multiple-choice questions), based on the core competencies for Thai PCU nurses [10] and in-house occupational health service frameworks [5]. Part 3 measured perceived self-efficacy (16 items, 5-point Likert scale: 5=extremely confident to 1=not at all confident), grounded in self-efficacy theory [8] and aligned with the same competency frameworks. Content validity was assessed by 3 experts from occupational health services, toxicology, and occupational health nursing curriculum, yielding a content validity index of 0.97. Reliability was measured using KR-20 for knowledge (0.94) and Cronbach’s α for professional attributes (0.71), skills (0.72), and perceived self-efficacy (0.94).

### Bias

Self-selection bias remains a concern due to voluntary participation. However, to minimize selection bias, the researchers tested the differences in baseline characteristics between the experimental and comparison groups, and found that the 2 groups were similar.

### Study size

Sample size was calculated using G*Power ver. 3.1 (Heinrich-Heine-Universität Düsseldorf), with a power of 0.80, a significance level of 0.05, and an effect size of 0.933 derived from a prior study’s mean and standard deviation (comparison group: 73.66±13.70; experimental group: 83.12±4.20) [9]. The calculated required sample size was 40 NPs. To account for potential dropout, an additional 50% was recruited, resulting in 60 participants, evenly divided between groups.

### Assignment method

The comparison group received basic patient care training from the Ministry of Public Health, identical to the training provided to the experimental group. After the study concluded, the comparison group received the SNPCOHS program.

### Blinding (masking)

Blinding was not implemented due to the study’s voluntary nature and feasibility constraints. Data analysis was performed independently at the group level to reduce bias.

### Unit of analysis

Data from all participants were analyzed by group.

### Statistical methods

This study used IBM SPSS ver. 20.0 (IBM Corp.) for data analysis. Descriptive statistics (mean, standard deviation, frequency, percentage) were calculated to describe general participant characteristics. Group comparisons of age, work experience post-NP training, and PCU experience were performed using the Mann-Whitney U test due to non-normal distributions. Gender, marital status, and highest education were compared using the Fisher exact test, as more than 20% had expected counts less than 5.

The data on professional attributes, knowledge, skills, and perceived self-efficacy of both groups were normally distributed according to the Shapiro-Wilk test (P>0.05). The independent t-test was used to analyze differences in the mean scores between the experimental and comparison groups at each time point. Two-way repeated measures analysis of variance was conducted to examine the mean score of professional attributes, knowledge, skills, and perceived self-efficacy of NPs in providing in-house services across 3 time points: baseline, post-intervention, and follow-up. The assumption of sphericity was tested using the Mauchly test. Results indicated a violation of the sphericity assumption for professional attributes (P≥0.05). Consequently, the Greenhouse-Geisser correction was applied, as the epsilon (ε) value was ≤0.750.

## Results

### Participants

The study included 60 NPs, with 30 participants in each group. Table 1 presents the demographic characteristics of each group. No statistically significant differences in baseline characteristics were observed between the groups (P>0.05).

### Main results

The mean scores for NPs’ competency, including occupational health service professional attributes, knowledge, and skills, and their perceived self-efficacy in delivering in-house occupational health services showed significant differences between the experimental and comparison groups across different time points (P<0.05). Within-group comparisons over time also showed significant changes in both groups (P<0.05) (Table 2).

In the experimental group, the mean scores for professional attributes, knowledge, skills, and perceived self-efficacy were significantly higher at post-intervention and follow-up than at baseline (P<0.05). However, no significant differences were observed between the scores at post-intervention and follow-up. In contrast, the comparison group showed no significant differences in any of the mean scores across time (Table 3).

The analysis of baseline data using the independent t-test revealed no significant differences between the experimental and comparison groups for any of the variables (P≥0.05). However, at both post-intervention and follow-up, the experimental group demonstrated significantly higher mean scores across all variables than the comparison group (P<0.05) (Table 4).

## Discussion

### Interpretation

The findings revealed meaningful improvements across 4 key domains—professional attributes, knowledge, skills, and perceived self-efficacy—among NPs providing in-house occupational health services. Specifically, participants demonstrated increased enthusiasm, leadership, and responsibility, indicating a positive shift in attitudes toward occupational health care. These improvements reflect a deeper recognition of nurses’ roles and a greater awareness of occupational risks among agricultural workers, particularly related to pesticide exposure.

In terms of knowledge, NPs gained substantial understanding of key occupational health concepts, such as pesticide safety, disease prevention, and integrating occupational health into routine nursing care. These advancements not only bolstered theoretical comprehension but also facilitated more accurate clinical documentation and informed decision-making, aligning clinical practices with academic and policy standards.

Skill development was evident in improved abilities for health promotion, inter-organizational collaboration, and effective communication regarding occupational risks. Nurses developed greater proficiency in coordinating with other sectors and conducting occupational health-related research, essential steps toward establishing comprehensive, community-based occupational health service models.

Perhaps most significantly, NPs reported increased confidence in delivering occupational health services. This increased self-efficacy suggests that training and practical experience empowered NPs to adopt more proactive roles, reinforcing the sustainable integration of occupational health services in primary care settings.

These changes are important because they directly support the expansion and effectiveness of occupational health services at the primary care level, in alignment with public health policies. They also reflect progress in building a workforce capable of addressing the complex needs of vulnerable working populations, ultimately contributing to the broader development of the occupational health system.

### Comparison with previous relevant studies

This study observed immediate and sustained improvements in professional attributes, knowledge, skills, and self-efficacy among NPs participating in the SNPCOHS program delivered via online learning. Unlike prior research indicating that onsite training did not significantly improve professional attributes [11], these findings suggest online learning—providing flexibility and repeated access—may better facilitate professional growth. Similarly, blended learning approaches combining e-learning with interactive elements like case studies and peer discussions have been more effective than traditional methods in enhancing nurses’ knowledge and skills [12]. Additionally, the increased self-efficacy observed aligns with previous studies highlighting the positive impact of social support from supervisors and colleagues on practitioners’ confidence [13].

### Limitations

The voluntary participation of NPs introduces the potential risk of self-selection bias, possibly affecting the generalizability of results. However, volunteers were not systematically different from non-volunteers regarding baseline occupational health knowledge, job responsibilities, or work locations. Additionally, current Ministry of Public Health policies in Thailand do not support occupational health training for primary care nurses, restricting such opportunities primarily to nurses in secondary or higher healthcare settings.

### Generalizability

The study’s limited timeframe and inclusion of participants from a single province may limit its generalizability to NPs across the broader national context.

### Suggestions

Outcomes (professional attributes, knowledge, skills) were not analyzed separately according to each of the 4 core competencies (interpersonal relationships, care management, integrated healthcare service, professional accountability). Future studies should assess outcomes separately for each competency to provide clearer evaluations.

### Implications

The SNPCOHS program can be effectively integrated into educational curricula to enhance occupational health competencies among healthcare personnel, especially in Southeast Asian countries or regions heavily reliant on agriculture and extensive pesticide use. Providing targeted education to healthcare workers, particularly nurses, can significantly improve occupational health services for agricultural workers exposed to pesticides at PCUs.

### Conclusion

The findings highlight the potential of the SNPCOHS program as a scalable model for enhancing occupational health competencies within PCUs. By equipping NPs with essential skills and confidence, the program addresses critical gaps in community-based occupational healthcare for underserved agricultural populations. The findings support investment in flexible, self-directed e-learning platforms as a strategy for strengthening public health workforces. Future research should explore sustainable and expansive interventions to facilitate long-term systemic improvements in occupational healthcare delivery.

## Figures and Tables

**Figure f4-jeehp-22-14:**
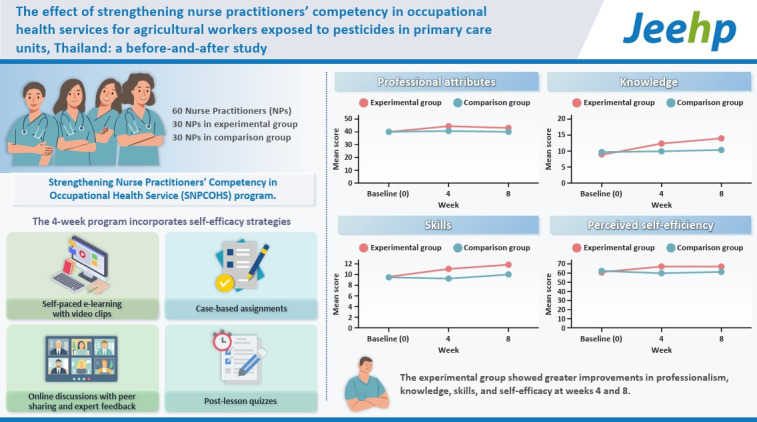


**Table 1.** Demographic characteristics of the experimental and comparison groups

Characteristic	Experimental group (n=30)	Comparison group (n=30)	P-value
Age (yr)	45.70±8.24	43.83±9.03	0.21^a)^
Gender			1.00^b)^
Male	1 (3.33)	1 (3.33)	
Female	29 (96.67)	29 (96.67)	
Marital status			0.51^b)^
Single	4 (13.33)	6 (20.00)	
Married	21 (70.00)	22 (73.33)	
Separate	5 (16.77)	2 (6.77)	
Highest education level			1.00^b)^
Bachelor’s degree	27 (90.00)	27 (90.00)	
Master’s degree	3 (10.00)	3 (10.00)	
Work experience after completing NP short course (yr)	9.67±4.87	7.87±5.42	0.21^a)^
Work experience in PCU (yr)	20.23±10.17	16.40±10.94	0.19^a)^

Values are presented as mean±standard deviation or number (%). NP, nurse practitioner; PCU, primary care unit.

a) By Mann-Whitney U test.

b) By Fisher exact test.

**Table 2.** Differences in mean scores according to group, time, and between groups across different time points

Variable	SS	df	MS	F	P-value
In-house occupational health services professional attributes of NPs^a)^					
Group	233.47	1	233.47	4.29	<0.05
Error (group)	1,579.36	29	54.46		
Time	166.80	1.48	112.81	20.51	<0.001
Error (time)	235.87	42.88	5.50		
Group*time	128.84	1.59	80.96	19.08	<0.001
Error (group*time)	195.82	46.15	4.24		
In-house occupational health services knowledge of NPs					
Group	131.76	1	131.76	21.36	<0.001
Error (group)	178.91	29	6.17		
Time	258.70	2	129.35	63.24	<0.001
Error (time)	118.63	58	2.05		
Group*time	152.14	2	75.07	36.41	<0.001
Error (group*time)	121.19	58	2.09		
In-house occupational health services skills of NPs					
Group	66.01	1	66.01	10.28	<0.05
Error (group)	186.16	29	6.42		
Time	53.73	2	26.87	6.07	<0.05
Error (time)	256.93	58	4.43		
Group*time	24.84	2	12.42	3.27	<0.05
Error (group*time)	220.49	58	3.80		
In-house occupational health services self-efficacy of NPs					
Group	616.05	1	616.05	3.89	<0.05
Error (group)	4,598.45	29	158.57		
Time	259.74	2	129.87	3.97	<0.05
Error (time)	1,859.26	58	32.68		
Group*time	810.70	2	405.35	15.26	<0.001
Error (group*time)	1,540.30	58	26.56		

SS, sum-of-squares; df, degrees of freedom; MS, mean square; NP, nurse practitioner.

a) P-value was calculated using the Greenhouse-Geisser method.

**Table 3.** Comparison of the mean differences in outcomes of the intervention group between baseline, week 4, and week 8

Data	Experimental group		Comparison group	
	MD±SD	P-value	MD±SD	P-value
In-house occupational health services professional attributes of NPs				
Week 4–baseline	4.200±0.466	<0.001	0.400±0.344	0.765
Week 8–baseline	3.267±0.589	<0.001	–0.670±0.650	1.000
Week 8–week 4	–0.933±0.335	0.280	–0.467±0.520	1.000
In-house occupational health services knowledge of NPs				
Week 4–baseline	3.433±0.525	<0.001	0.333±0.211	0.374
Week 8–baseline	5.100±0.497	<0.001	0.700±0.236	0.018
Week 8–week 4	1.667±0.366	<0.001	0.367±0.242	0.422
In-house occupational health services skills of NPs				
Week 4–baseline	1.367±0.497	0.031	–0.130±0.400	1.000
Week 8–baseline	2.167±0.800	0.015	0.500±0.469	0.886
Week 8–week 4	0.800±0.537	0.441	0.633±0.471	0.567
In-house occupational health services self-efficacy of NPs				
Week 4–baseline	6.900±1.573	<0.001	–3.000±1.151	0.043
Week 8–baseline	6.733±1.262	<0.001	–0.967±1.549	1.000
Week 8–week 4	–0.167±1.287	1.000	2.033±1.550	0.599

Adjustment for multiple comparisons using the Bonferroni method. MD, mean difference; SD, standard deviation; NP, nurse practitioner.

**Table 4.** Differences in outcomes between the experimental and comparison groups

Data	Experimental group		Comparison group		P-value
	Mean±SD	Min–max	Mean±SD	Min–max	
In-house occupational health services professional attributes of NPs					
Baseline	40.00±3.16	32–48	40.10±4.49	30–50	0.921
Week 4	44.20±3.66	35–50	40.50±4.99	32–51	0.002
Week 8	43.27±3.89	33–50	40.03±5.77	27–49	0.014
In-house occupational health services knowledge of NPs					
Baseline	9.03±1.75	6–13	9.80±1.47	7–13	0.072
Week 4	12.47±1.99	9–16	10.13±1.48	7–12	<0.001
Week 8	14.13±1.98	9–17	10.50±1.36	8–12	<0.001
In-house occupational health services skills of NPs					
Baseline	9.60±2.39	3–14	9.43±2.27	5–15	0.783
Week 4	10.97±2.62	4–15	9.30±1.75	6–13	0.006
Week 8	11.77±2.64	4–16	9.93±2.70	5–15	0.010
In-house occupational health services self-efficacy of NPs					
Baseline	60.23±7.14	41–71	62.40±8.40	46–78	0.286
Week 4	67.13±5.41	57–78	59.40±9.91	43–80	<0.001
Week 8	66.97±7.66	48–80	61.43±9.41	35–80	0.015

By independent t-test. SD, standard deviation; NP, nurse practitioner.

## Footnotes

**Data availability**
Data files are available from Harvard Dataverse: https://doi.org/10.7910/DVN/F4IY8V Dataset 1. The response data from participants.

## Supplementary materials

Supplementary files are available from Harvard Dataverse: https://doi.org/10.7910/DVN/F4IY8V

Supplement 1. The detail and content of the strengthening nurse practitioners’ competency in the occupational health service program.

Supplement 2. Audio recording of the abstract.
