# Supplementary material for: The effect of strengthening nurse practitioners’ competency in occupational health services for agricultural workers exposed to pesticides in primary care units, Thailand: a before-and-after study
Source: J Educ Eval Health Prof. 2025 Apr 21;22:14. doi: 10.3352/jeehp.2025.22.14 (PMC12138529; doi:10.3352/jeehp.2025.22.14)
Supplement: Supplementary file 2 — Supplement 1. The detail and content of the strengthening nurse practitioners’ competency in the occupational health service program. [file jeehp-22-14-suppl1.docx]

**Supplement 1.** The detail and content of the strengthening nurse practitioners’ competency in the occupational health service program.

The activity of strengthening NPs’ competency on occupational health services at PCUs for agricultural workers exposed to pesticides program a total of 10 hours (0.67 credits).

1. Self-study on online lessons (e-learning) and clip video 5 hours.

2. Online discussion (sharing information, the experts provide verbal explanations, and sharing their own occupational health services) 3 hours.

3. Assignments (case study and video clip creation) 2 hours.

| **Week** | **Module** | **Topic** | **Concept** | **Activity form self-efficacy theory based on four primary sources of information** |
| --- | --- | --- | --- | --- |
| 1^st^ | 1 | Fundamental of pesticides. | Knowledge (Care management)  Knowledge (Integrated healthcare service) | - Self-study on online lessons (e-learning) and video clips (Enactive mastery experience)  - Evaluating post-test after self-study on online lessons (Emotional arousal) |
|  | 2 | Principles of occupational health, occupational hazards, and health surveillance for agricultural workers exposed to pesticides. | Traits (Care management)  Traits (Integrated healthcare service)  Knowledge (Interpersonal relationship)  Knowledge (Care management)  Knowledge (Integrated healthcare service)  Knowledge (Professional accountability)  Skills (Care management)  Skills (Integrated healthcare service) | - Self-study on online lessons (e-learning) and video clips (Enactive mastery experience)  - Evaluating post-test after self-study on online lessons (Emotional arousal)  - Online discussion, sharing information (Enactive mastery experience)*  - The experts provide verbal explanations (Verbal persuasion)*  - Case study (Enactive mastery experience) |
|  | 3 | Various laws related to providing in-house occupational health services. | Traits (Professional accountability)  Knowledge (Care management)  Knowledge (Integrated healthcare service) | - Self-study on online lessons (e-learning) and video clips (Enactive mastery experience)  - Evaluating post-test after self-study on online lessons (Emotional arousal) |
| 2^nd^ | 4 | Health promotion and prevention of occupational diseases and related strategies. | Traits (Interpersonal relationship)  Traits (Professional accountability)  Knowledge (Interpersonal relationship)  Knowledge (Integrated healthcare service) | - Self-study on online lessons (e-learning) and video clips (Enactive mastery experience)  - Evaluating post-test after self-study on online lessons (Emotional arousal)  - Online discussion, sharing information (Enactive mastery experience)* |
|  | 5 | Screening for occupational diseases for agricultural workers exposed to pesticides. | Knowledge (Integrated healthcare service)  Skills (Integrated healthcare service) | **-** Self-study on online lessons (e-learning) and video clips (Enactive mastery experience)  - Evaluating post-test after self-study on online lessons (Emotional arousal)  - Online discussion, sharing information (Enactive mastery experience)*  - Case study (Enactive mastery experience) |
|  | 6 | Basic first aid and referral for agricultural workers exposed to pesticides | Knowledge (Integrated healthcare service)  Skills (Integrated healthcare service) | - Self-study on online lessons (e-learning) and video clips (Enactive mastery experience)  - Evaluating post-test after self-study on online lessons (Emotional arousal)  - Case study (Enactive mastery experience)  - Online discussion, sharing information (Enactive mastery experience)*  - Sharing their own occupational health services after participating in an e-learning program (Emotional arousal)* |
| 3^th^ | 7 | Primary medical care for agricultural workers exposed to pesticides. | Knowledge (Care management)  Knowledge (Integrated healthcare service)  Skills (Integrated healthcare service) | - Self-study on online lessons (e-learning) and video clips (Enactive mastery experience)  - Evaluating post-test after self-study on online lessons (Emotional arousal)  - Online discussion, sharing information (Enactive mastery experience)*  - Case study (Enactive mastery experience) |
|  | 8 | Recording service results and data management. | Knowledge (Care management)  Skills (Care management) | - Self-study on online lessons (e-learning) and video clips (Enactive mastery experience)  - Evaluating post-test after self-study on online lessons (Emotional arousal)  - Online discussion, sharing information (Enactive mastery experience)*  - Sharing their own OH services after participating in an e-learning program (Emotional arousal)*  - Case study (Enactive mastery experience)  - Video clip creation (Enactive mastery experience) |
| 4^th^ | 9 | Risk communication and health literacy to providing in-house occupational health services for agricultural workers exposed to pesticides. | Knowledge (Interpersonal relationship)  Skill (Interpersonal relationship) | - Self-study on online lessons (e-learning) and video clips (Enactive mastery experience)  - Evaluating post-test after self-study on online lessons (Emotional arousal) |
|  | 10 | Role of nurses in providing in-house occupational health services and fundamentals nursing research. | Traits (Interpersonal relationship)  Traits (Care management)  Traits (Integrated healthcare service)  Traits (Professional accountability)  Knowledge (Professional accountability  Skills (Professional accountability | - Self-study on online lessons (e-learning) and video clips (Enactive mastery experience)  - Evaluating post-test after self-study on online lessons (Emotional arousal)  - Online discussion, sharing information (Enactive mastery experience)*  - Sharing experiences (living model) and demonstration video clips (symbolic modeling) (Vicarious experience)*  - The experts provide verbal explanations (Verbal persuasion)*  - Case study (Enactive mastery experience) |

**Note:** * Organize activities in week 4

- Participants could switch between any modules, in weeks 1-2, at least 3 modules per week need to be studied, and in weeks 3-4, at least 2 modules per week.
- Developing one concept can involve a variety of activities, while skills development focuses on doing case studies, online discussion, sharing information and demonstration video clips.
